# Supplementary material for: The Effect of Mealworm Frass on the Chemical and Microbiological Properties of Horticultural Peat in an Incubation Experiment
Source: Int J Environ Res Public Health. 2022 Dec 20;20(1):21. doi: 10.3390/ijerph20010021 (PMC9819234; doi:10.3390/ijerph20010021)
Supplement: Supplementary file 1 [file ijerph-20-00021-s001.zip › ijerph-2067751-supplementary.pdf]

Table S1. Selected chemical properties of mealworm (*Tenebrio molitor* L.) frass and deacidified peat.

Parameter	Frass	Deacidified peat
pH (distilled H_2O)	5.60	5.70
EC (mS cm^{-1})	6.92	0.16
C (g kg^{-1} DM)	388.10	457.25
N-total (g kg^{-1} DM)	38.00	14.69
P (g kg^{-1} DM)*; P (mg dm^{-3} DM)**	14.70	25.90
K (g kg^{-1} DM)*; K (mg dm^{-3} DM)**	19.70	75.41
Mg (g kg^{-1} DM)*; Mg (mg dm^{-3} DM)**	4.27	69.35

* – total nutrient content (P, K and Mg) in frass; ** – content of available nutrients (P, K and Mg) in deacidified peat

Table S2. Quantitative Polymerase Chain Reaction Set-up.

Target gene	Primer name	Set-up	Reference
Bacterial load	BAC338F<br>BAC805R<br>BAC516F (probe)	Initial denaturation at 95°C for 10 min, followed by 42 cycles at 95°C for 15 s and 60°C for 1 min.	Yu et al. [31]
Clostridium spp.	CI-F1<br>CI-R2<br>Probe-I (probe)	Initial denaturation at 95°C for 10 min, followed by 45 cycles at 95°C for 20 s, at 63°C for 30 s, and at 72°C for 45 s.	Song et al. [32]
Pseudomonas spp.	B2BF<br>B2BR3	Initial denaturation at 95°C for 30 s, followed by 40 cycles with a 5-s denaturation step at 95°C and a 34-s elongation/extension step at 60°C, and ending with a melt curve analysis at 95°C for 15 s, 60°C for 1 min, and 95°C for 15 s.	Hu et al. [33]
Fungal load	FungiQuant-F<br>FungiQuant-R<br>FungiQuant-Prb (probe)	UNG treatment at 50°C for 3 min, initial denaturation at 95°C for 10 min, followed by 50 cycles at 95°C for 15 s and 65°C for 1 min.	Liu et al. [34]
Bacillus spp.	16SBACF<br>16SBACR	Initial denaturation at 95°C for 10 min, followed by 45 cycles at 95°C for 15 s, at 58°C for 55 s, and at 72°C for 30 s. Melting curve analyses were performed as follows: denaturing step at 95°C for 15 s, annealing at 60°C for 1 min, and melting in steps of 0.3°C up to 95°C for 15 s.	Mora et al. [35]
Ammonium monooxygenase (*amoA*)	amoA-1F<br>amoA-2R	Initial denaturation at 95°C for 10 min, followed by 40 cycles at 95°C for 30s, 55°C for 30 s, and 72°C for 45 s.	Rotthauwe et al. [36]
Nitrogenase reductase (*nifH*)	nifH-F<br>nifH-Rb	Initial denaturation at 95°C for 10 min, followed by 40 cycles: at 95°C for 15 s, at 60°C for 30 s, and 72°C for 40 s.	Rösch and Bothe [37]
Nitrite reductase (*nirS*)	nirS cd3AF<br>nirS R3cd	Initial denaturation at 95°C for 10 min, followed by 40 cycles: at 95°C for 15 s, at 58°C for 30 s, and 72°C for 40 s.	Michotey et al. [38], Throbäck et al. [39]
Nitrous oxide reductase (*nosZ*)	nosZ-F<br>nosZ-R	Initial denaturation at 95°C for 10 min, followed by 40 cycles: at 95°C for 15 s, at 62°C for 30 s, and 72°C for 40 s.	Kloos et al. [40] Throbäck et al. [39]
Urease (*ureC*)	ureC1F<br>ureC2R	Initial denaturation at 95°C for 10 min, followed by 40 cycles: at 95°C for 15 s, at 60°C for 1 min, and 72°C for 2 min.	Koper et al. [41]
